# Early CD8^+^-recovery independently predicts low probability of disease relapse but also associates with severe GVHD after allogeneic HSCT

**DOI:** 10.1371/journal.pone.0204136

**Published:** 2018-09-20

**Authors:** Juha Ranti, Samu Kurki, Urpu Salmenniemi, Mervi Putkonen, Soile Salomäki, Maija Itälä-Remes

**Affiliations:** 1 Department of Hematology and Stem Cell Transplantation Unit, Division of Medicine, Turku University Hospital, Turku, Finland; 2 Auria Biobank, University of Turku and Turku University Hospital, Turku, Finland; University of Kentucky, UNITED STATES

## Abstract

In this single-center study we retrospectively evaluated the impact of early reconstitution of different lymphocyte subsets on patient outcomes after allogeneic hematopoietic stem cell transplantation (allo-HSCT). We found that CD8^+^ T-cell counts exceeding 50x10^6^/l as early as on day 28 post-transplantation correlated significantly with decreased relapse risk, with three-year relapse rates of 17.0% and 55.6% (P = 0.002), but were also associated with severe acute and chronic GVHD. Incidence of grade III-IV acute GVHD was 30.5% for those with early CD8^+^ T-cell recovery compared to 2.1% for those with lower CD8^+^ T-cell counts on day 28 post-transplant (HR = 20.24, P = 0.004). Early CD8^+^ T-cell reconstitution did not, however, affect the overall survival. Multivariate analysis showed that slow CD8^+^ T-cell reconstitution was strongly associated with increased risk of relapse (HR = 3.44, P = 0.026). A weaker correlation was found between CD4^+^ reconstitution and relapse-risk, but there was no such association with CD19^+^ B-cells or NK-cells. In conclusion, the early CD8^+^ T-cell recovery on day 28 post-transplant is associated with the lower risk of relapse but also predicts the impending severe GVHD, and thus could be useful in guiding timely treatment decisions.

## Introduction

Allogeneic hematopoietic stem cell transplantation (allo-HSCT) is an intensive treatment modality, which offers a potential cure for many malignant and non-malignant hematological disorders. The main drawback of allo-HSCT is the significant risk of transplant related mortality (TRM), mainly due to the graft-vs-host disease (GVHD) and severe infections [1–3]. TRM has been decreasing during the last years by the introduction of less intensive conditioning regimens and better supportive care, and is now reported to have a frequency of around 15–20% from previous figures of 30–40% in the 1980s and 1990s [4]. Along with the decrease in TRM, disease relapse has become the leading cause of death after transplantation [5].

Lymphocytes play a major role in GVHD as well as in graft-versus-leukemia (GVL) reactions [6]. After allo-HSCT, lymphocytes recover after proliferation of the myeloid compartment, and different subsets of immune cells reconstitute further at different schedules. NK-cell reconstitution is relatively fast and occurs within 30–100 days. On the other hand, adaptive immunity, which requires functional T- and B-lymphocytes, takes considerable longer time to recover: T-cells reconstituting about 100 days after transplantation and B-cell reconstitution taking up to 1–5 years [7].

Early after transplantation, T-cell reconstitution consists of expansion of donor-derived memory-type CD45^+^RO^+^ T-cells, which have been infused with the allogeneic stem cell graft. Later in the post-transplantation period, T-cell immune reconstitution relies on *de novo* production of naïve CD45^+^RA^+^ T-cells in the recipient’s thymus. These newly produced T-cells originate from lymphoid progenitors arising from the donor's hematopoietic stem cells [8]. Slow recovery of T-lymphocytes predisposes the recipient to opportunistic infections, but obviously also to other adverse events as low lymphocyte counts have been shown to be associated with poor clinical outcome in general [9–11].

The aim of this study was to evaluate the influence of reconstitution of different lymphocyte subsets on clinical outcome, with special emphasis on the association between CD8^+^ T-cell recovery and the relapse rate. We hypothesized that early reconstitution of CD8^+^ T-cells might be associated with better outcome after transplantation considering their role in GVL reactions and direct cytotoxic effects against various pathogens [12].

## Materials and methods

### Patients

During the study period between January 2013 and July 2016, 170 patients received an allo-HSCT at the Turku University Hospital, Finland. Blood lymphocyte subsets (CD3^+^, CD8^+^, CD4^+^, CD19^+^, CD16^+^) were measured monthly by flow cytometry. After exclusion of 50 patients due to the incomplete lymphocyte data sets, 120 patients with complete data sets were included in the study.

The patients were classified into five groups by their diagnoses: Group 1 patients with myeloid malignancies; acute myeloid leukemia (AML) and myelodysplastic syndrome with excess of blasts (MDS-RAEB); Group 2 lymphatic malignancies: acute lymphoblastic leukemia (ALL) and lymphoblastic lymphoma (LBL); Group 3 myeloproliferative diseases: chronic myeloid leukemia (CML), chronic myelomonocytic leukemia (CMML), primary myelofibrosis (PMF), polycythemia vera (PV) and essential thrombocythemia (ET); Group 4 lymphoproliferative diseases: chronic lymphocytic leukemia (CLL), multiple myeloma (MM), and lymphomas; and Group 5 severe aplastic anemia (SAA).

Disease stage was defined according to the disease risk index (DRI) [13]. EBMT risk score was used for evaluation of transplant risk [14].

This retrospective study was approved by Institutional Review Board of Turku University Hospital and all patients had given their informed written consent in accordance with Declaration of Helsinki for registry data analysis. None of the transplant donors were from a vulnerable population and all donors or next of kin provided written informed consent that was freely given.

### Conditioning

All conditioning regimens used in this study are described in detail in previous publications [15–17]. Myeloablative conditioning (MAC; n = 33) consisted mainly of fludarabine 125 mg/m^2^ combined with busulfan 12.8 mg/kg (FluBu4), or of high-dose cyclophosphamide 120 mg/kg and high-dose busulfan 12.8 mg/kg (BuCy), or (in patients with ALL) of high-dose cyclophosphamide 120 mg/kg in combination with total body irradiation at a total dose of 10–12 Gy. Reduced intensity conditioning (RIC; n = 57) mainly consisted of fludarabine combined with busulfan (FluBu2 or FluBu3; Bu 3.2 mg/kg for two or three days). Sequential conditioning (n = 30) consisted of an induction with a combination of fludarabine, high-dose cytarabine and idarubicin or amsacrine, followed by a conditioning with cyclophosphamide (80–120 mg/kg) and TBI 4 Gy (FLAMSA-RIC).

GVHD prophylaxis consisted of a short course of methotrexate, rabbit-derived anti-thymocyte globulin (ATG; Thymoglobulin®, Sanofi Genzyme) 2.5 mg/kg on days -2 and -1, calcineurin inhibitor tacrolimus and mycophenolate mofetil (MMF) on days 1–30. In haploidentical transplantations, GVHD prophylaxis included post-transplant high-dose cyclophosphamide (50mg/kg on days +3 and +4), low-dose ATG (as above), MMF and tacrolimus. G-CSF was not used during the post-transplantation period.

### Monitoring of minimal residual disease, chimerism and infectious agents

The presence of minimal residual disease (MRD) was followed by real-time qPCR-based methods whenever suitable PCR probes were available. Multiparameter flow cytometry and *fluorescence in situ* (FISH) were used in those lacking a PCR probe. MRD was assessed by analysis of bone marrow samples drawn every 2 to 3 months over the first two years and thereafter when needed. Lymphomas were followed by PET-CT or CT at three month intervals until three years post-transplant.

Donor chimerism was monitored by alleleSEQR technique (Abbott, Chicago, IL, USA). In alleleSEQR -analysis 34 biallelic insertion/deletion loci across the entire human genome were screened to identify one recipient specific and two donor specific markers. The real-time PCR was performed with the Applied Biosystems® 7500 multicolor PCR system (Thermo Fisher, Waltham, MA, USA) as recommended by the manufacturer and Abbott AlleleSEQR software was used to analyze the real-time PCR data.

Cytomegalovirus (CMV) and Epstein-Barr-virus (EBV) were monitored weekly by qPCR over the first three months post-transplant, and thereafter if the patient had ongoing immunosuppression.

### Diagnosis and treatment of GVHD

Diagnosis and grading of aGVHD and cGVHD were based on clinical and histopathological findings [18–19]. Chronic GVHD was classified as mild, moderate or severe according to the NIH criteria [20]. Mesenchymal stem cells were used for steroid refractory aGVHD.

### Preemptive treatment of imminent relapse

Preemptive treatment of imminent relapse consisted of early withdrawal of immunosuppression, azacytidine with or without donor lymphocyte infusions (DLI), lenalidomide, FLT3-inhibitors or ibrutinib. Preemptive treatment was guided by MRD and/or chimerism analysis.

### Data source

Lymphocyte laboratory measurements (CD3^+^ T-cells, CD4^+^ T-cells, CD8^+^ T-cells. CD19^+^ B-cells and CD16^+^ NK-cells) were queried from the hospital data warehouse through the means of big data analysis. This included 2075 lymphocyte subset analyses in total and an average of 17.3 lymphocyte subset measurements per patient. All other clinical variables were retrieved from the electronic patient reports. The follow-up period ended in November 2016.

Lymphocyte measurements were selected for each patient at time points of 1, 3 and 6 months after allotransplantation in a window of +- 7 days. Surviving patients with missing lymphocyte data at any of these time points during the follow-up were excluded from the final analysis. Receiver operating characteristics (ROC) analysis was performed for these three time points and each lymphocyte laboratory descriptor was analyzed to predict post-transplant relapse and mortality.

### Clinical endpoints

The relapse free survival (RFS) was analyzed by using time from transplantation to relapse. Relapse was defined as MRD-positivity of previously MRD-negative patient. Overall survival was analyzed by using the time from transplantation to death due to any cause. Disease-free survival was analyzed by using time from transplantation to relapse or death due to any cause, whichever came first. Patients without events were censored at the last day of the follow-up.

### Statistics

Relapse rate, overall survival and disease-free survival were studied using Kaplan-Meier analysis and log rank test. ROC analysis for the highest Youden’s index (J = sensitivity + specificity– 1) was performed to find cut-off values for laboratory measurements to predict post-transplant relapse and mortality. Cox’s regression analysis was used to calculate univariate and multivariate hazard ratios, 95% confidence intervals and P-values for each variable to predict post-transplant relapse. Comparisons between groups were performed using one-way ANOVA for continuous variables and χ^2^-test for categorical variables. All P-values were two-sided, and P-values < 0.05 were considered statistically significant.

## Results

### Patient characteristics

Baseline patient characteristics are described in detail in Table 1. The most common diagnoses were myeloid malignancies, *i*.*e*. AML and MDS-RAEB (42.5%). The median duration of follow-up for surviving patients at the end of the study was 1.6 (range 0.3–3.5) years. MAC, RIC or sequential (FLAMSA-RIC) conditioning regimens were used in 27.5%, 47.5%, and 25.0% of the patients, respectively. Most of the grafts had been collected from peripheral blood (83.3%), fewer from bone marrow (16.7%). None of the grafts were manipulated. The median EBMT risk score for study patients was 3 (range 1–6).

**Table 1:** The baseline characteristics of the study’s patients.

Variable		N or median	% or range
Gender	Female	56	46.7
	Male	64	53.3
Age at transplantation (years)		55.1	17.0–69.0
Diagnosis	Myeloproliferative neoplasms	16	13.3
	Myeloid malignancies	51	42.5
	Lymfoproliferative neoplasms	38	31.7
	Lymphatic malignancies	10	8.3
	Aplastic anemia	5	4.2
Donor	MUD	91	75.8
	HLA-identical	23	19.2
	Haploidentical	6	5
Graft CD34^+^ cell count (x10^6^/kg)		5.7	1.0–15.6
Graft source	Blood	100	83.3
	Marrow	20	16.7
Conditioning	MAC	33	27.5
regimen	RIC	57	47.5
	Sequential conditioning	30	25
DRI stage	Low	79	65.8
	High	36	30
	Not applicable	5	4.2
EBMT risk score		3	1.0–6.0
Relapses		27	22.5
Deaths		36	30
Follow-up time (years)		1.6	0.3–3.5

The patient characteristics of the excluded 50 patients are provided as a supplementary material (S1 Table). Regarding these characteristics, there were no statistically significant differences between the patients included and excluded from the study.

### CD8^+^ T-cell cut-off values

Samples for lymphocyte analyses were collected for each patient at 1, 3 and 6 months after allo-HSCT. ROC analysis was performed for these three time points and for each lymphocyte descriptor to predict relapse after the allo-HSCT. The highest Youden’s index was obtained with CD8^+^ T-cell count using a cut-off value of 50x10^6^/l at one month post-transplant. Hence, the CD8^+^ T-cell count at 1 month was used as a variable in the analyses to predict the relapse risk.

For the analyses of early recovery of CD8^+^ T-cells, the patients were divided into two groups based on their CD8^+^ T-cell count on day 28 post-transplant. The CD8^+^_high28_–group were those patients who had early recovery of CD8^+^ T-cells and had a CD8^+^-count of >50x10^6^/l, while the CD8^+^_low28_–group were those patients who had late recovery of CD8^+^ T-cells and had a CD8^+^-count of <50x10^6^/l on day 28. Seventy-two patients (60% of the all patients) reached the CD8^+^ T-cell count of 50x10^6^/l by the day 28 (+- 7 days) post-transplant, and were referred to the early recovery group (CD8^+^_high28_–group).

### Overall survival (OS) and disease-free survival (DFS)

**Fig 1:**
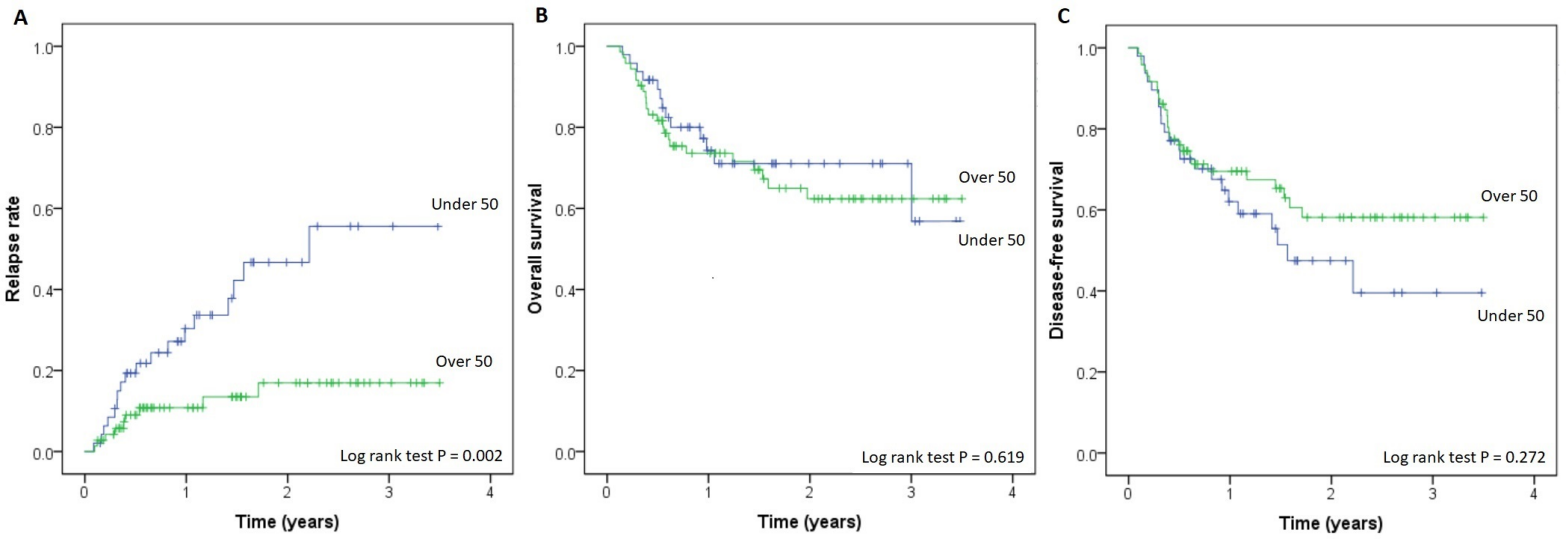
A) relapse-rate, B) overall survival and C) disease-free survival in study’s patients represented as Kaplan-Meier curves according to CD8^+^ T-cell status at one month.

There was a trend towards longer disease-free survival in the CD8^+^_high28_-group, but it did not reach statistical significance (P = 0.272) (Fig 1C). Overall survival did not differ between the two patient groups (three-year survival 62.4% vs. 56.8%, P = 0.619). We also carried out a separate ROC analysis to find an optimal cut-off value for early CD8^+^ T-cell reconstitution correlating with longer survival, but due to weak association between CD8^+^-counts and OS, no such cut-off could be found (S1 Fig).

We also analyzed the significance of the higher CD8^+^ T-cell count on day 90 post-transplant, with the CD8^+^ T-cell cut-off value of 400x10^6^/l, which has been shown to correlate with better transplant outcomes in the haploidentical transplant setting [21]. In our study, however, there was no difference in OS or relapse rates between CD8^+^_high90_- and CD8^+^_low90_-groups with this cut-off value (data not shown).

### Effect of CD8^+^ T-cell recovery on mortality

Thirty-six patients died during the follow-up period (overall mortality 30%). Twenty-three of these patients (63.9%) belonged to the CD8^+^_high28_-group and 13 patients (36.1%) to the CD8^+^_low28_-group. Subgroup-specific mortality was not significantly different in the two groups, 32.0% (23 patients) in the CD8^+^_high28_-group and 27.1% (13 patients) in the CD8^+^_low28_-group (HR = 0.79, P = 0.570).

The causes of death varied significantly between the two groups. Disease relapse was the main cause of death in the CD8^+^_low28_-group (69.2% of deaths, 9 patients), but not in the CD8^+^_high28_-group (26.1% of deaths, 6 patients), HR = 6.38, P = 0.016. In contrast, GVHD-related mortality was higher in the CD8^+^_high28_-group compared to the CD8^+^_low28_-group, 34.8% (8 patients) vs. 7.7% of deaths (1 patient), respectively (HR = 6.40, P = 0.100). Mortality due to infection was slightly more common in the CD8^+^_high28_-group compared to the CD8^+^_low28_-group, 34.8% (8 patients) and 23.1% (3 patients) of deaths (HR = 1.48, P = 0.467), respectively.

### Relapse rate

The relapse rate was significantly lower in the CD8^+^_high28_-group (Fig 1A, three-year rate 17.0% vs. 55.6%, P = 0.002).

We used several variables, as patient age, donor type, graft source or intensity of conditioning in univariate and multivariate analyses to find out any associations with disease relapse (Table 2). The slow CD8^+^-lymphocyte reconstitution was a strong predictive factor for disease relapse by both univariate (HR = 3.33, P = 0.003) and multivariate analysis (HR = 3.44, P = 0.026). In multivariate analysis the high disease stage assessed by the DRI (HR 3.197, P = 0.018) was also independently correlated with increased relapse frequency. The presence of moderate or severe GVHD was, in turn, linked with decreased risk of relapse (HR = 0.288, P = 0.041).

**Table 2:** The univariate and multivariate analysis by Cox regression regarding factors affecting the relapse-risk after an allogeneic stem cell transplantation.

	Univariate			Multivariate		
Variable	HR	95% CI	Pvalue	HR	95% CI	P value
Gender (Male)	0.65	0.356–1.188	0.161	0.385	0.151–0.982	**0.046**
Age at transplantation > 55.1 years (median)	1.009	0.986–1.032	0.466	1.006	0.963–1.051	0.798
One month CD8 (under 50x10^6^/l)	3.328	1.49–7.431	**0.003**	3.441	1.156–10.242	**0.026**
Diagnosis (Myeloproliferative neoplasms)	1	Reference category		1	Reference category	
Diagnosis (Myeloid malignancies)	0.758	0.34–1.69	0.499	2.431	0.625–9.464	0.2
Diagnosis (Lymfoproliferative neoplasms)	0.406	0.161–1.023	0.056	1.088	0.217–5.446	0.918
Diagnosis (Lymphatic malignancies)	0.935	0.312–2.801	0.904	3.306	0.39–28.01	0.273
Diagnosis (Aplastic anemia)	0.845	0.182–3.919	0.83	2.093	0.166–26.409	0.568
Donor (MUD)	1	Reference category		1	Reference category	
Donor (HLA-identical)	0.907	0.417–1.972	0.806	2.325	0.722–7.488	0.157
Donor (Haploidentical)	2.293	0.696–7.552	0.172	1.843	0.229–14.823	0.566
Transplant cell count (<5.7x10^6^/kg, median)	1.015	0.92–1.121	0.763	0.953	0.794–1.145	0.608
Transplant type (Bone marrow)	1.292	0.599–2.786	0.514	1.080	0.248–4.697	0.919
Treatment (Myeloablative conditioning)	1	Reference category		1	Reference category	
Treatment (Reduced-intensity conditioning)	1.068	0.488–2.337	0.868	2.730	0.51–14.625	0.241
Treatment (Sequential)	2.049	0.926–4.535	0.077	2.542	0.515–12.538	0.252
DRI stage (High)	2.663	1.206–5.879	**0.015**	3.197	1.225–8.339	**0.018**
Acute GVHD (Grade II to Grade IV)	1.057	0.489–2.284	0.887	2.301	0.869–6.094	0.094
Chronic GVHD (Moderate to Severe)	0.262	0.098–0.697	**0.007**	0.288	0.088–0.95	**0.041**

We also analyzed the effect of other lymphocyte subsets’ recovery on the relapse risk (S2 Fig and S2 Table). According to the ROC analysis, there was a correlation between decreased relapse risk and the early recovery of CD3^+^- and CD4^+^-lymphocytes. However, the ability of early CD8^+^-recovery to predict decreased relapse risk surpassed that of the CD3^+^- and CD4^+^-cells. The recovery rate of CD19^+^ B-cells and CD16^+^ NK-cells had no effect on the relapse rate.

Regarding the late post-transplant lymphocytosis, our data was too scarce to show any association between lymphocytosis and decreased relapse rate (data not shown).

### Association between acute or chronic GVHD and early versus late CD8^+^ T-cell recovery

The incidence of acute GVHD grade II-IV was more common in the CD8^+^_high28_-group compared to the CD8^+^_low28_-group, 56.9% and 23.4%, respectively (HR = 4.33, P<0.001). In accordance, incidence of severe grade III-IV aGVHD was higher in the CD8^+^_high28_-group, 30.5% vs. 2.1% (HR = 20.24, P = 0.004).

Early CD8^+^ T-cell reconstitution was also associated with an increased incidence of chronic GVHD. Moderate or severe chronic GVHD occurred more often in the CD8^+^_high28_-group (48.6% vs. 16.7%, HR = 4.73, P = 0.001). Most of the patients (75%) in the slowly recovering CD8^+^ T-cell group developed no or only mild chronic GVHD. Furthermore, the incidence of scleroderma-type skin cGVHD was more common in the CD8^+^_high28_-group (12.5% vs. 2.1%, HR = 6.71, P = 0.076).

### Kinetics of post-transplant CD8^+^ T-cell count recovery

**Fig 2:**
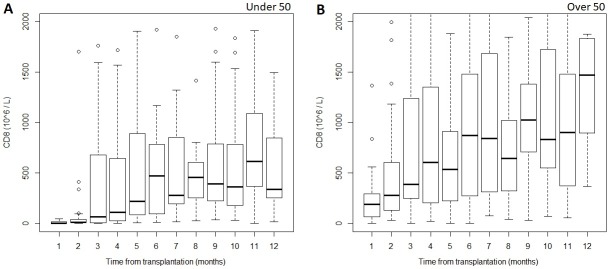
CD8^+^ T-cell levels measured within the first year after transplant in patients with A) one month CD8^+^-count below 50x10^6^/l and B) above 50x10^6^/l.

In the CD8^+^_low28_-group CD8^+^ T-cell reconstitution began slowly, CD8^+^ T-cell counts reaching the laboratory set reference values (190-1140x10^6^/l) only after 6 months post-transplant, and staying at the lower end of the reference range to the end of the first year (Fig 2). In contrast, the median CD8^+^ T-cell count in the CD8^+^_high28_-group reached the reference range within two months post-transplant, and reached and even exceeded the upper level of reference range by 12 months post-transplant, as shown in Fig 2B.

Thus, the progression of CD8^+^ T-cell reconstitution can be predicted very early after transplantation. The median CD8^+^-cell count was significantly higher in the CD8^+^_high28_-group compared to the CD8^+^_low28_-group at all selected timepoints: at 1 month (190x10^6^/l vs. 4x10^6^/l, P = 0.001), 3 months (387.5x10^6^/l vs. 64.5x10^6^/l, P = 0.040), 6 months (872.5x10^6^/l vs. 474.5x10^6^/l, P = 0.053) and 12 months (1474.5x10^6^/l vs. 337x10^6^/l, P = 0.013) post-transplant.

### Factors affecting CD8^+^ T-cell reconstitution

We analyzed the effect of some clinical variables on the CD8^+^ T-cell reconstitution by multivariate analysis at two timepoints, at 1 and 6 months post-transplant (Table 3). The pre-transplant diagnosis was associated with the rate of CD8^+^ T-cell reconstitution. Higher levels of CD8^+^ T-cell counts were reached on day 180 in the patients transplanted for AML or MDS-RAEB than in the patients transplanted for myeloproliferative neoplasms or lymphatic malignancies, with medians of 1062x10^6^/l (range 90-4557x10^6^/l), 259x10^6^/l (range 15-689x10^6^/l) and 345.5x10^6^/l (range 136l-3898x10^6^/l), respectively (p = 0.01). Early CD8^+^ T-cell reconstitution is independently associated with higher CD8^+^ T-cell counts at 6 months post-transplant (p = 0.006).

**Table 3:** Clinical variables affecting the CD8^+^-reconstitution at one and six months after allotransplantation.

CD8^+^ T-cell recovery		One month			Six months		
Variable		Median	Range	P-value	Median	Range	P-value
Gender	Female	93.3	0–3270	0.401	505	15–4557	0.507
	Male	85	0–1388		604.5	3.5–3371	
Age at HSCT	Under 55.1 (median)	85	0–3270	0.85	494	28–3898	0.16
(years)	Over 55.1	89.5	0–2666		533	3.5–4557	
One month CD8^+^	Under 50x10^6^/l	6.5	0–48	**0**	272.5	15–3011	**0.006**
	Over 50x10^6^/l	191.5	52–3270		920	3.5–4557	
Diagnosis	Myeloproliferative neoplasms	78	1–595	0.654	259	15–411	**0.01**
	Myeloid malignancies	158	0–858		1062	90–4557	
	Lymfoproliferative neoplasms	63.8	0–2666		653	3.5–2188.5	
	Lymphatic malignancies	67.8	1–3270		345.5	136–3898	
	Aplastic anemia	13	1–814		264.5	61–586	
Donor	Matched unrelated donor	85	0–3270	0.729	619.5	3.5–4557	0.057
	HLA-identical	137	0–1375		478.3	58–1606	
	Haploidentical	7.3	2–261		104.5	27–153.5	
Transplant CD34^+^	Under 5.7x10^6^/l (median)	150.8	0–3270	0.21	493.3	15–3898	0.179
cell count	Over 5.7x10^6^/l	54	0–2666		810	3.5–4557	
Graft source	Blood	87.3	0–3270	0.321	604.5	3.5–4557	0.169
	Marrow	31.5	0–814		264.5	15–3727.5	
Conditioning	Myeloablative conditioning	67	0–3270	0.673	434	28–3898	0.613
regimen	Reduced intensity conditioning	97	0–1388		671	3.5–3870.5	
	Sequential conditioning	64.5	1–828		562.8	27–4557	
DRI stage	Low	99	0–3270	0.336	659.3	4–3898	0.768
	High	85	0–1388		364	28–4557	
Acute GVHD	Grade 0 to Grade I	45	0–3270	0.904	710.8	15–4557	0.214
	Grade II to Grade IV	115.5	1–1375		411	4–3871	
Chronic GVHD	Mild		n/a		543.5	4–3011	0.127
	Moderate to Severe		n/a		510.7	15–4557	

At 6 months post-transplant, there was a trend towards statistically significantly lower CD8^+^ T-cell counts in patients receiving haploidentical transplants (median 104.5x10^6^/l, P = 0.057). The source of stem cell graft or conditioning regimen did not have impact on the late CD8^+^ T-cell recovery. Regarding the early CD8+ T-cell reconstitution, there were no clinical variables associated with higher CD8^+^ T-cell counts at 1 month post-transplant.

## Discussion

We evaluated recovery of the lymphocyte subsets from monthly taken blood samples in patients who had received allo-HSCT for hematological disorders from HLA-matched unrelated, sibling or haploidentical donors. However, due to the low number of haploidentical allografts evaluated in this study, the conclusions are best applied to HLA-matched allotransplantation. We found that late recovery of the CD8^+^ T-cell subset was the strongest independent predictor of disease relapse. Regarding the other lymphocyte subsets, the CD4^+^ T-cell recovery rate was weakly associated with relapses and CD19^+^ B-cell or NK-cell reconstitution carried no such association. ROC analysis was used to find the optimal time point and the best cut-off value for the CD8^+^ T-cell count. We found that even a slight increase in the CD8^+^ T-cell count to the level of 50x10^6^/l, as early as on day 28 post-transplant, was strongly associated with a reduced relapse rate.

Of note, in contrast to most previous reports, the graft source (PBSC or BM) or intensity of conditioning regimen did not impact the relapse risk in the present study [22,23]. The lack of correlation between intensity of the conditioning regimen and the relapse risk is most obviously caused by the high proportion of sequential conditioning. Sequential conditioning was used also in the patients with a responsive disease, but with high relapse risk due to the high pre-transplant MRD level. Intensity of the conditioning (MAC v RIC) did not correlate to the relapse risk, which might be explained by the fact that almost all patients with lymphoma received RIC conditioning with rather good outcomes. The low number of bone marrow derived grafts probably contributed to the lack of association between graft source and relapse risk.

The lower relapse rate in the CD8^+^_high28_-group was achieved at the expense of a higher frequency of both acute and chronic GVHD. This was not unexpected, as the CD8^+^ T-cells play an important role in GVHD, and GVHD may, at least in part, be based on the same alloreactivity as GvL reactions [6, 24–25]. Early reconstitution of CD8^+^-lymphocytes seems to be a strong predictive factor for the most severe forms of acute and chronic GVHD, and early CD8^+^ T-cell reconstitution might serve as an indication for more intensive immunosuppressive therapy for preventing forthcoming GVHD, but this needs to be confirmed in further studies.

The rate of post-transplant lymphocyte recovery has been reported to impact on the recipient's prognosis in various trials. Michelis et al. showed that an absolute total lymphocyte count (ALC) above 0,5 x 10^9^/l on day 28 post-transplant is associated with decreased relapse risk and better 3-year OS in AML patients [26]. Kim et al. found that low ALC in the first three months after transplantation is a significant predictive factor of poor OS and higher NRM [10]. The correlation of early lymphocyte reconstitution and early recovery of lymphocyte function with improved transplant outcomes has been corroborated also in the other previous studies [27–29]. Furthermore, an early rise in the ALC and absolute monocyte count has also been reported to be associated with significantly better OS in the context of PBSC, BM, and cord blood derived grafts [30]. A rapid rise of CD4^+^ T-cell count after transplantation has been reported, as well, to be correlated with better OS and lower TRM [31].

To our knowledge, there are no previous studies reporting an association between delayed CD8^+^ T-cell reconstitution and a higher relapse rate among patients who have received an allo-HSCT from a matched unrelated or HLA-identical sibling donor. In the present study, there was a trend towards better DFS in the CD8^+^_high28_-group though it did not reach statistical significance (P = 0.27, Fig 2C). Early recovery of the CD8^+^ T-cell subset correlated inversely with relapse risk, but the positive effect of fewer relapses was counteracted by increased GVHD-related mortality. An early rise in the total lymphocyte count has been shown to be associated with better transplant outcomes, but in these studies the CD8^+^ T-cell count has not had an independent prognostic impact [10].

In the haploidentical setting, Tian et al. showed that reaching the CD8^+^ T-cell count 375x10^6^/l by the day 90 post-transplant was associated with lower NRM and longer LFS [21]. In their study, higher CD8^+^ T-cell counts three months post-transplant had a major positive impact on survival, mainly due to a lower risk of infections, but the rate of relapses or GVHD were not influenced by the CD8^+^ T-cell counts. In the present study, there was no difference in OS or relapse rates between CD8^+^ T-cell counts above or under 400x10^6^/l on day 90 in the cohort of the patients transplanted with HLA-matched unrelated or sibling donor. The causes for differing results between these two studies may include the different conditioning regimens and the use of post-transplant G-CSF potentially leading to distinct immune reconstitution in the haploidentical setting. The missing influence of CD8^+^ recovery at 3 months compared to the greater impact at 1 month in our study might be explained by the lymphocyte lowering effect of aGVHD and corticosteroid treatment, making the evaluation of lymphocyte counts more difficult later during the post-transplant period.

The use of ATG may decelerate immune reconstitution after allotransplantation, but since all the patients were treated with ATG this was not a confounding factor in the present study [32]. Furthermore, treatment with glucocorticoids is also known to delay immune reconstitution, as are reportedly the use of a HLA mismatched donor, lower CD34^+^-cell count in the graft and bone marrow derived graft [10, 26, 33]. We evaluated several clinical variables and their influence on CD8^+^ T-cell reconstitution, and none of them correlated significantly with faster CD8^+^ T-cell reconstitution at the time point of 28 days post-transplant, although there was a slight trend towards faster CD8^+^ T-cell recovery in patients with higher CD34^+^-cell dose in the graft (P = 0.20).

Regarding late CD8^+^ T-cell recovery, we found that patients receiving the graft from a HLA-matched unrelated donor or sibling donor, compared to haploidentical donor, tended to have higher CD8^+^ T-cell counts at 6 months after transplantation (P = 0.057). Slower immunological reconstitution following transplantation from a haploidentical donor has been reported in the previously as well [34, 35]. Patients with diagnosis of AML or MDS had higher CD8^+^ T-cell counts at 6 months, though the diagnosis did not have any influence on the early recovery of CD8^+^ T-cells. It has been shown, that the thymic recovery starts to influence T-lymphocyte values around 6 months after alloHSCT. It is therefore surprising, that older age or myeloablative conditioning, often associated slower thymic recovery, did not carry any association with late CD8^+^ T-cell reconstitution [36]. Moreover, it is known, that GVHD slows down thymic recovery after alloHSCT as well, but in the end CD8^+^ T-cell counts were significantly higher through the first year among the more GVHD susceptible CD8^+^_high28_-group. The lacking effect of these suppressors of thymic recovery on late CD8^+^-counts can be explained by the notion, that the CD8^+^-recovery after alloHSCT relies more on the peripheral expansion than T-cell production from naïve thymocytes [7].

## Conclusions

According to our findings, the early recovery of CD8^+^ T-cells is inversely associated with risk of relapse and directly associated with a risk of severe acute and chronic GVHD, regardless of diagnosis, intensity of conditioning regimen or graft source. As TRM has been decreasing in the recent years, disease relapse has become the leading cause of death after allo-HSCT. We found that a cut-off value of 50x10^6^/l CD8^+^ T-cells at 1 month after transplantation is a powerful predictive factor of forthcoming relapse after an HLA-matched allograft. Thus, the CD8^+^ T-cell count on day 28 post-transplant is a simple and feasible indicator of the risk level of disease relapse, potentially offering guidance on timing of prophylactic or preemptive post-transplant approaches.

## Supporting information

S1 Table The baseline characteristics of the excluded patients. (XLSX)

S2 Table The AUC values and optimal cutoff points for different lymphocyte subsets correlating with the increased relapse risk. (XLSX)

S1 Fig **The ROC curves regarding the correlation between the early CD8**^**+**^**-recovery and a) the relapse risk and b) overall survival.** Regarding association between the rate of CD8^+^-recovery and relapse rate, a clear cut off could be found. In contrast, regarding the OS, ROC analysis revealed no significant correlation with CD8^+^-recovery and overall survival.
(TIF)

S2 Fig The ROC analysis regarding the different lymphocyte subsets’ recovery in correlation with the relapse risk. (TIF)
