# Supplementary figures and images for: Early CD8+-recovery independently predicts low probability of disease relapse but also associates with severe GVHD after allogeneic HSCT
Source: PLoS One. 2018 Sep 20;13(9):e0204136. doi: 10.1371/journal.pone.0204136 (PMC6147489; doi:10.1371/journal.pone.0204136)

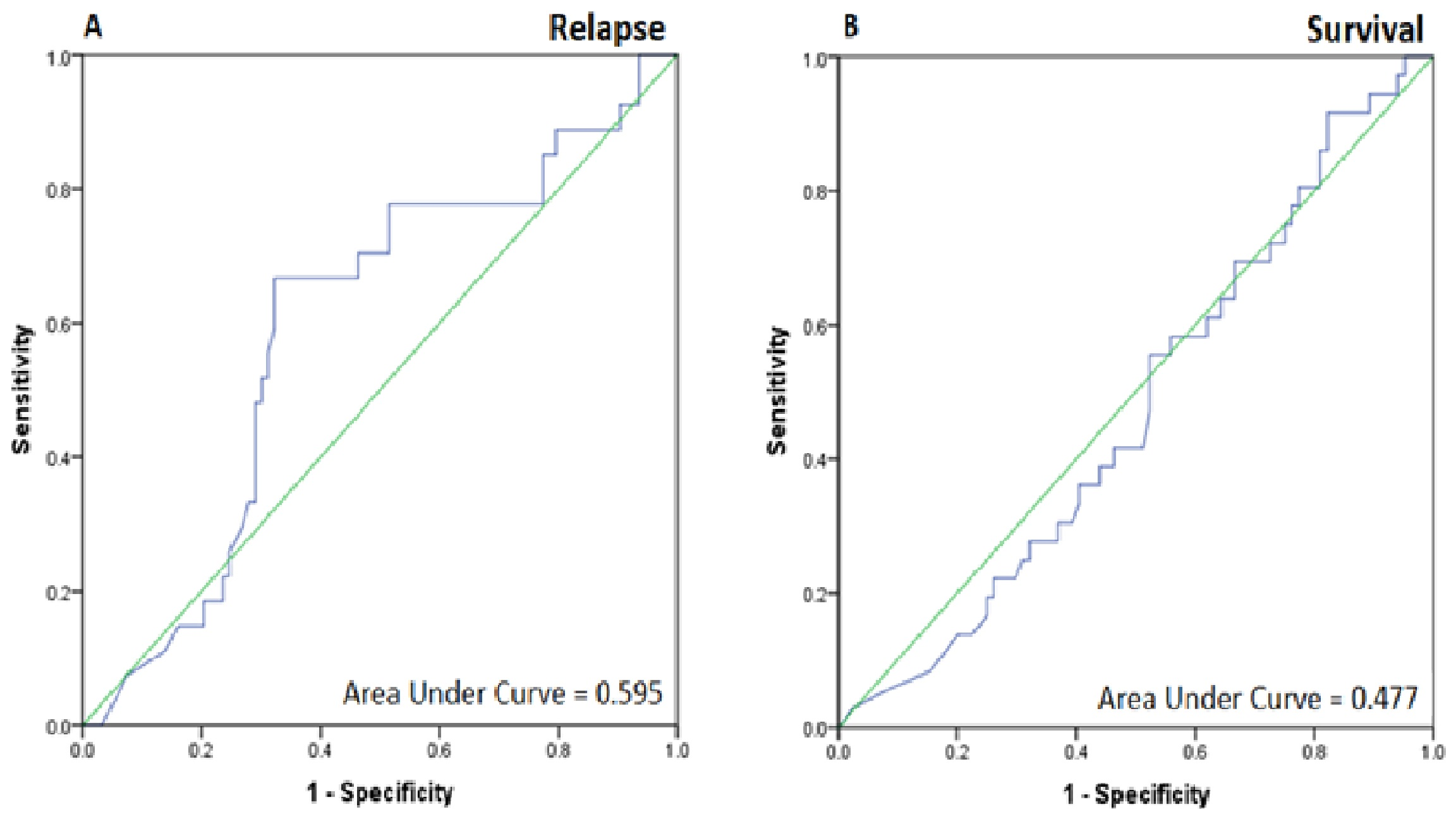

Supplement: S1 Fig — The ROC curves regarding the correlation between the early CD8+-recovery and a) the relapse risk and b) overall survival. Regarding association between the rate of CD8+-recovery and relapse rate, a clear cut off could be found. In contrast, regarding the OS, ROC analysis revealed no significant correlation with CD8+-recovery and overall survival. (TIF) [file pone.0204136.s003.tif]

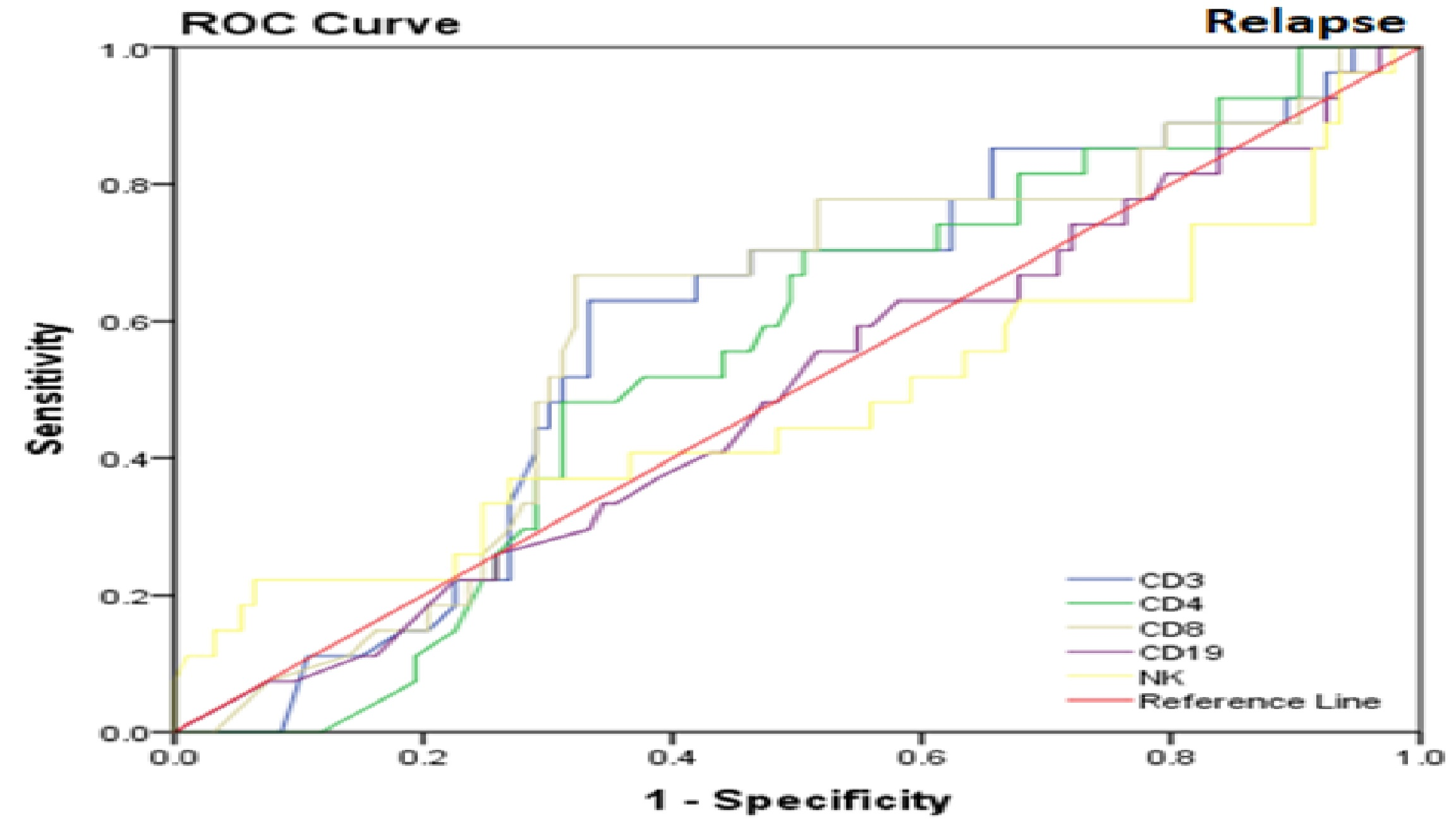

Supplement: S2 Fig — (TIF) [file pone.0204136.s004.tif]
